# Dynamics of Bacterial Diversity in Fish Farming Lagoons: Implications for the Ecosystem Trophic Status

**DOI:** 10.3390/biology14111563

**Published:** 2025-11-07

**Authors:** María Custodio, Richard Peñaloza

**Affiliations:** 1 Environmental Science & Health Research Group, Facultad de Medicina Humana, Universidad Nacional del Centro del Perú, Av. Mariscal Castilla N° 3909, Huancayo 12006, Peru; rpenhaloza@uncp.edu.pe; 2 Facultad de Zootecnia, Universidad Nacional del Centro del Perú, Av. Mariscal Castilla N° 3909, Huancayo 12006, Peru

**Keywords:** bacterial diversity, 16S rRNA, sediment microbiome, aquaculture, Andean lagoons, nitrogen, chlorophyll-*a*

## Abstract

Bacterial communities in lake sediments are key to ecosystem health and fish productivity, yet little is known about their composition in the high-altitude Andean lagoons of central Peru. In aquaculture systems, these microbial assemblages regulate nutrient recycling, organic matter degradation and oxygen availability, forming a key component of the microbial loop. Intensive trout farming introduces a continuous nutrient load through uneaten feed and metabolic byproducts, which fuels eutrophication and reshapes benthic microbial structure. This study evaluated the bacterial diversity and community structure in sediments from four fish farming lagoons (Pomacocha, Habascocha, Tipicocha, and Trancagrande). Environmental variables, including dissolved oxygen (DO), inorganic nitrogen, inorganic phosphorus, and chlorophyll-a, were measured to determine trophic status. Sediment bacterial composition was analyzed using Illumina sequencing of 16S rRNA gene amplicons, and community structure differences were assessed with diversity indices and SIMPER analysis. Microbial patterns were assessed at phylum and class levels to capture changes across taxonomic scales. Pseudomonadota, Actinobacteria, Cyanobacteria and Bacteroidetes dominated across lagoons, with significant among-site variation. Richness was similar, but Tipicocha showed the highest evenness (Shannon H′ = 2.769; Simpson 1-D = 0.8969). SIMPER identified Deltaproteobacteria, Alphaproteobacteria, Actinobacteria and Gammaproteobacteria as major contributors to dissimilarity. The presence of Methanomicrobia and Bacilli in Tipicocha and Trancagrande indicated micro-oxic to anoxic conditions, characteristic of systems with high organic load and redox heterogeneity. These results show that aquaculture alters trophic status and reshapes sediment bacterial communities in high-Andean lagoons. Understanding these patterns is essential to design monitoring and management strategies that balance aquaculture productivity with the conservation of sensitive ecosystems.

## Simple Summary

This study addressed the impact of fish farming on Peru’s high Andean lakes, key ecosystems for environmental health and fishery productivity. The problem is that uneaten food and fish waste introduce large amounts of nutrients, altering water quality and the bacterial communities present in the sediments. The objective of this study was to evaluate bacterial diversity and structure in four lakes. The results showed that, although species richness was similar, bacterial composition varied significantly, with Lake Tipicocha being the most diverse. The presence of bacteria indicative of high organic matter content and low oxygen levels is clear evidence that the organic load from fish farming is altering the chemistry of the lagoon bottoms. The conclusion is that fish farming modifies the nutritional status and microbial diversity of these sensitive lagoons. This knowledge is essential for designing strategies that allow fish farming without compromising the conservation of these important Andean ecosystems.

## 1. Introduction

Fish production in temperate lagoons is constantly expanding globally in response to growing demand for protein sources [1,2,3]. In 2020, continental fish farming accounted for approximately 56% of global aquaculture production [4]. Despite its growth, much of the knowledge about the environmental footprint of this practice comes from comprehensive studies in the marine salmon farming industry.

Scientific evidence has revealed that aquaculture generates a significant load of organic waste and nutrients. It is estimated that between 70% and 88% of the inorganic nitrogen and phosphorus in feed is deposited on the lake bottom [5,6]. In addition, waste derived from feeding (feces and metabolic excretions from farmed species) significantly deteriorates water quality in farming systems [7,8]. The nutrient input from fish production could alter the properties of sediments and, consequently, affect the bacterial communities responsible for driving the biogeochemical cycles that directly influence nutrient availability, water quality, and the ability of ponds to sustain aquatic life [9,10].

Several studies have shown how fish farming can drastically alter the trophic status of water bodies, increasing nutrient loads and modifying the physicochemical conditions of sediment [7,11]. Consequently, this impacts the structure of sedimentary bacterial communities, favoring the proliferation of bacterial groups associated with the degradation of organic matter and conditions of hypoxia or anoxia, displacing native and beneficial microbial communities. Studies in low-altitude marine and freshwater ecosystems have consistently reported an increase in the abundance of Gammaproteobacteria and Deltaproteobacteria under culture cages [12]. These groups are involved in the degradation of complex organic matter and in anaerobic cycles such as sulfate reduction, processes that intensify in enriched anoxic sediments [13]. Therefore, these changes can have negative repercussions on ecosystem functionality, compromising its resilience and the sustainability of fish farming itself [14,15].

Understanding the impact of these factors that shape the aquatic microbiome is essential to understanding how they influence the health of the aquatic system. To address this complexity and obtain a detailed characterization of the bacterial communities, environmental DNA (eDNA) analysis has been consolidated as a precise molecular technique [16,17]. This approach offers the necessary resolution to rigorously evaluate the impact of fish farming on bacterial structure [18,19].

Fish farming is a rapidly expanding economic activity in Latin America, particularly in lacustrine ecosystems in Colombia, Peru, and Brazil. This practice introduces contaminants such as uneaten feed, metabolic waste, and pharmaceuticals, generating an excessive load of nutrients (notably nitrogen) in the water and sediments [20,21,22]. However, the adaptation of this industry to high-altitude ecosystems with unique limnological characteristics poses a critical ecological uncertainty. Despite its relevance, there is a marked lack of knowledge about the composition and diversity of bacterial communities in the sediments under the pressure of Andean fish farming. Without this information, it is impossible to understand the real ecological impact and develop management strategies adapted to the sensitivities of these high-mountain ecosystems.

The research problem focuses on the lack of data that correlate the intensification of fish farming in Peruvian high-Andean lagoons with the alteration of their sedimentary bacterial communities, a key indicator of ecosystem health. In this context, continuous monitoring of aquatic environmental quality is essential for understanding ecological conditions and improving environmental management [23]. The main objective of this study was to evaluate the bacterial composition and diversity in sediment from four lagoons (Pomacocha, Habascocha, Tipicocha, and Trancagrande) under pressure from fish farming in the central region of Peru. The specific objectives were (a) to determine environmental variables using analytical methods and the trophic status of the water, (b) to analyze bacterial composition and diversity using Illumina sequencing, and (c) to establish the correlation between environmental variables, trophic status, and the assembly of bacterial communities present in the sediments. Ultimately, the findings of this research are crucial for understanding the impact of fish farming on Peruvian high-Andean aquatic ecosystems. This information is essential for guiding management and monitoring strategies that ensure the sustainability of fish farming and the conservation of these lacustrine systems.

## 2. Materials and Methods

### 2.1. Study Area

**Figure 1:**
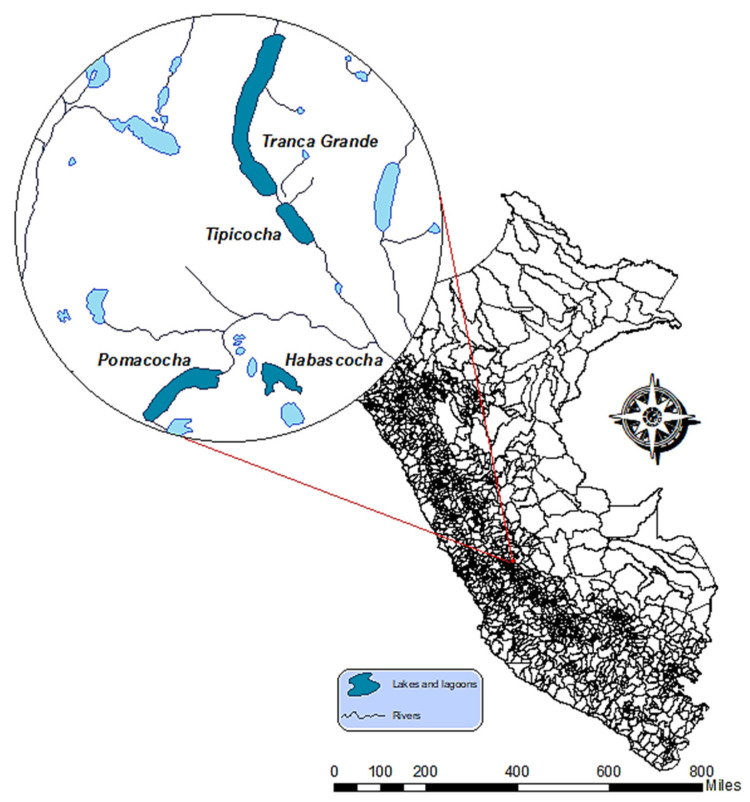
Location map of the study area.

The study was conducted in four high-altitude glacial lakes located in the Perené River basin in the central Andes of Peru: Pomacocha, Habascocha, Tipicocha, and Trancagrande. These lagoons, located between 4310 and 4330 m above sea level, are known for their use in intensive rainbow trout (*Oncorhynchus mykiss*) farming, which is carried out in floating cage systems. The high altitude of these lakes makes them unique and sensitive ecological environments, where aquaculture activity can have a significant impact on water quality and local biodiversity. These high-Andean lagoons are situated in a region characterized by unique geological formations and a climate heavily influenced by seasonal variations in precipitation and drought. Their hydrology is critically dependent on snowmelt, glacial retreat, rainfall, and subterranean seepage, which are vital for maintaining water levels and regional water security. The ecological state is further influenced by local anthropogenic activities, including agriculture, fish farming and livestock grazing, contributing to nutrient loading and potential eutrophication [24]. The specific location of the water bodies, which are located between latitudes −11.7808° and −11.7198° and longitudes −75.2454° and −75.2311°, places them in a region of ecological and economic importance (Figure 1).

### 2.2. Field Measurements and Sample Collection

#### 2.2.1. Water Sampling and Analysis

Three sampling stations were established per lake, with three samples collected at each station during November 2019. Dissolved Oxygen (DO) was determined in situ using a WTW Multi3630 IDS multi-parameter instrument, and chlorophyll-*a* (Chl-*a*) was measured with an Aquaprobe AP5000 Fluorometer. For chemical analysis, water samples were collected in 500 mL glass bottles. After collection, they were transported to the laboratory and stored at 4 °C until preparation and analysis.

Concentrations of inorganic nitrogen and phosphorus were analyzed at the Water Research Laboratory of the National University of Central Peru, using a Lovibond MD600 photometer. All analyses were performed in triplicate, and the results were reported as the mean value. All analyses followed the procedures described in the Standard Methods for the Examination of Water and Wastewater [25].

#### 2.2.2. Sediment Sampling and Quality Control

Sampling points were established strategically near areas with aquaculture activity. Composite samples of surface sediment (0–10 cm depth), approximately 250 g, were collected in each lake using a stainless-steel auger-type device. To ensure sample integrity and minimize cross-contamination between the lakes (following standard practices), Quality Control measures were adopted. These included strict cleaning of the auger-type device within situ water from the destination lake and a subsequent rinse with distilled water before each new collection. Finally, the samples were transferred to sterile and airtight plastic bags, cold-preserved (on ice or refrigerated/frozen depending on the specific subsequent analysis), and immediately transported to the Molecular Biology Laboratory of the National University of Tumbes for processing.

### 2.3. DNA Extraction

Genomic DNA was extracted from 0.5 g of homogenized sediment using the Presto™ Soil DNA Extraction Kit, following the manufacturer’s instructions. DNA concentration and purity were assessed using a NanoDrop™ One spectrophotometer (Thermo Fisher Scientific, Waltham, MA, USA), yielding values between 0.3 and 88.5 ng/µL.

### 2.4. PCR Amplification and 16S rRNA Sequencing

The detailed characterization of the bacterial communities was performed using environmental DNA (eDNA) analysis and high-throughput sequencing of the 16S rRNA gene [18], an essential approach to overcome the limitations of traditional culture methods [16,17]. PCR amplification of the V3–V4 hypervariable regions of the 16S rRNA gene (generating a product of approximately 1365 bp) was performed using universal primers 27F (5′-AGAGTTTGATCCTGGCTCAG-3′) and 1392R (5′-GGTTACCTTGTTACGACTT-3′). The reaction mix was prepared in a total volume of 25 µL, combining 1 µL of each primer, 22 µL of master mix (buffer, dNTPs, and Taq polymerase) and 1 µL of DNA template, 1 µL of 27F primer, 1 µL of 1392R primer and 22 µL of a PCR mix (containing premix buffer, dNTPs, and Taq polymerase), using Gene One and GE Healthcare Life Sciences kits. Amplification was carried out using a scheme validated in microbiome studies for the amplification of the 1365 bp 16S rRNA region. The cycle began with an initial denaturation at 95 °C for 5 min to ensure complete separation of the template DNA. This was followed by 35 repeated cycles of denaturation at 95 °C for 30 s, annealing at 55 °C for 30 s, and extension at 72 °C for 90 s. Finally, complete transcription of the copies was ensured with a final extension at 72 °C for 7 min. Sequencing was conducted using the Illumina MiSeq v2 platform, following the standard paired-end protocol [26,27,28,29]. Library preparation and sequencing were outsourced to Admera Health LLC. (South Plainfield, NJ, USA).

### 2.5. Bioinformatic Processing of Sequencing Data

Raw paired-end reads were quality-filtered and processed using QIIME2 (version 2023.5). Chimeric sequences were identified and removed using the DADA2 plugin, and high-quality amplicon sequence variants (ASVs) were taxonomically assigned against the SILVA 138 database at 97% similarity. Rarefaction curves were generated to confirm sequencing depth and sample coverage before diversity analyses.

### 2.6. Data Analysis

#### 2.6.1. Trophic Index (TRIX)

The TRIX was used to assess the trophic status of the lagoons in this study. It is a multimetric tool derived from the combination of inorganic nitrogen, inorganic phosphorus, dissolved oxygen, and Chl-*a* [30]. The general formula for calculating TRIX is:

(1) $$TRIX=\frac{{log}_{10}(Chl\text{-a}\times \left|D\%O_{2}\right|\times DIN\times DIP)}{m}+k$$

where Chl-*a* represents the concentration of Chl-*a*, D%O_2_ represents the percentage deviation in absolute value of the oxygen concentration from saturation conditions (in %). DIN and DIP represent the concentration of dissolved inorganic nitrogen and dissolved inorganic phosphorus, respectively. The parameters k = 1.5 (lower limit) and m = 1.2 (scale adjustment) are normalization constants that allow the index to oscillate within a predefined numerical range, generally between 0 and 10. Higher TRIX values indicate a higher level of eutrophication. According to Vollenweider et al., values close to 0 indicate low eutrophication, while values close to 10 indicate high eutrophication [31] (Table 1).

#### 2.6.2. Statistical Analysis

Descriptive and inferential statistical analyses were performed to characterize the structure of the microbial community in the lagoons. Data generated from physicochemical variables and diversity indices were tested for normality using the Shapiro–Wilk test and for homogeneity of variance using Levene’s test. Alpha diversity was assessed using indices such as taxonomic richness (S), Shannon (H′), Simpson (1-D), dominance (D), and Chao-1 estimated richness. Physicochemical parameters (DO, DIN, DIP and Chl-*a*) were analyzed using the same assumption tests, and because normality and homoscedasticity criteria were not consistently met, differences among lagoons were evaluated using the non-parametric Kruskal–Wallis test followed by Dunn’s post hoc comparisons with Bonferroni correction. As the preliminary tests indicated that the parametric assumptions were not fully satisfied, differences in alpha diversity indices and physicochemical variables among the four lagoons were tested using the non-parametric Kruskal–Wallis H test, followed by Dunn’s post hoc test with Bonferroni correction for pairwise comparisons. Statistical analyses were performed using the R software (v 4.3.1) [32] with the Vegan (V 2.7-1) and ggplot2 (V 4.0.0) packages, considering a statistical significance level of *p* < 0.05. Beta diversity was explored using Bray–Curtis distance matrices. To identify the bacterial taxa that contributed most to the differences in community composition between the lagoons, a similarity percentage analysis (SIMPER) based on Bray–Curtis dissimilarity was performed. Taxa were classified as ‘dominant contributors’ if their individual contribution to the total dissimilarity exceeded 5.0%, and as ‘minor contributors’ if their individual contribution was between 2.0% and 5.0% and deemed ecologically relevant. The analysis was performed using square root-transformed relative abundance data of bacterial taxa (phylum and class levels) derived from the 16S rRNA gene sequencing dataset. In addition, SIMPROF (similarity profile analysis) clustering was used to detect statistically significant differences. Ecological interactions were assessed using Spearman’s rank correlation to link microbial abundances with environmental variables, retaining only significant results (*p* < 0.05).

## 3. Results

### 3.1. Physicochemical Characteristics and Trophic Status of Lagoons

Dissolved oxygen (DO) levels were highest in Habascocha (12.193 ± 0.686 mg/L) and decreased progressively toward Trancagrande (9.850 ± 0.597 mg/L), with significant differences among lagoons (*p* < 0.001). All DO values were above the minimum threshold established by the U.S. Environmental Protection Agency for aquatic systems [33]. Inorganic nitrogen concentrations varied significantly, ranging from 0.122 ± 0.010 mg/L in Habascocha to 0.153 ± 0.017 mg/L in Tipicocha, and remained below the EPA guideline of 0.3 mg/L for freshwater ecosystems [33]. Inorganic phosphorus concentrations did not differ significantly among lagoons, with values between 0.008 ± 0.001 mg/L and 0.009 ± 0.002 mg/L (Table 2), all below the recommended limit of 0.05 mg/L for lakes and reservoirs. Chlorophyll-a concentrations were highest in Tipicocha (0.020 ± 0.001 mg/L), differing significantly from the remaining lagoons. The integrated TRIX classified all lagoons as eutrophic (TRIX > 5.4), with Tipicocha registering the highest value (5.739 ± 0.081).

### 3.2. Taxonomic Composition at the Phylum Level

The phylum Pseudomonadota dominated all lagoons, representing the highest number of sequences in each site (66,426 in Tipicocha and 64,971 in Trancagrande). This phylum was consistently the most abundant group across all lagoons. Cyanobacteria was the second most abundant phylum in Pomacocha (20,855) and Trancagrande (22,762), with comparatively lower counts in Habascocha and Tipicocha. In contrast, Actinobacteria was especially abundant in Habascocha (20,234) and showed a marked reduction in Trancagrande (3407).

Other phyla detected in all systems included Bacteroidetes, Firmicutes, Chloroflexi, and Acidobacteria, each exhibiting variable read numbers across lagoons. Archaeal lineages such as Euryarchaeota and Thaumarchaeota were present in all sites, with higher sequence counts in Tipicocha and Trancagrande. Additional phyla such as Gemmatimonadetes, Planctomycetes, and Verrucomicrobia were consistently present with moderate reads. Low-abundance phyla—including Chrysiogenetes, Crenarchaeota, and Elusimicrobia—appeared sporadically, each representing fewer than 10 reads in most lagoons (Table 3).

### 3.3. Alpha Diversity of Bacterial Classes

The four Andean lagoons exhibited comparable bacterial class richness, with values ranging from 62 to 65 taxa (Table 4). This total includes Candidatus lineages, which are recognized based on genetic evidence but lack formal taxonomic characterization. Tipicocha registered slightly lower richness than Habascocha and showed the highest values for the Shannon and Simpson indices. These metrics indicate relatively high evenness and low dominance (D = 0.1031) within its bacterial community. In contrast, Habascocha and Trancagrande exhibited higher dominance values. Pomacocha showed intermediate richness and evenness. Estimated richness based on the Chao-1 index closely matched observed richness in all lagoons, confirming sufficient sequencing depth and low representation of undetected taxa at the class level.

### 3.4. Community Dissimilarity and Key Contributors at the Class Level

The SIMPER analysis identified the bacterial classes contributing most substantially to community dissimilarities in surface sediments. In the comparison between Habascocha and Pomacocha, the dominant contributors were Deltaproteobacteria (18.2%) and Alphaproteobacteria (18.1%), followed by Actinobacteria (9.2%) and Gammaproteobacteria (8.5%). These four classes accounted for more than half of the total compositional dissimilarity (54%). The additional presence of Flavobacteriia and Betaproteobacteria raised the cumulative contribution to 68%.

In the Habascocha–Tipicocha comparison, Actinobacteria (23.4%), Betaproteobacteria (13.3%), and Deltaproteobacteria (13.0%) were the main contributors. Methanomicrobia (5.0%) also appeared in this comparison. For Habascocha vs. Trancagrande, Actinobacteria (20.3%) and Deltaproteobacteria (12.6%) again ranked among the top contributors, followed by Alphaproteobacteria and Gammaproteobacteria. Bacilli and Methanomicrobia were also detected as minor contributors (Table 5). Overall, SIMPER analysis revealed consistent differences in the relative contribution of dominant bacterial classes among lagoons.

### 3.5. Environmental Gradients and Distribution Patterns at the Phylum Level

**Figure 2:**
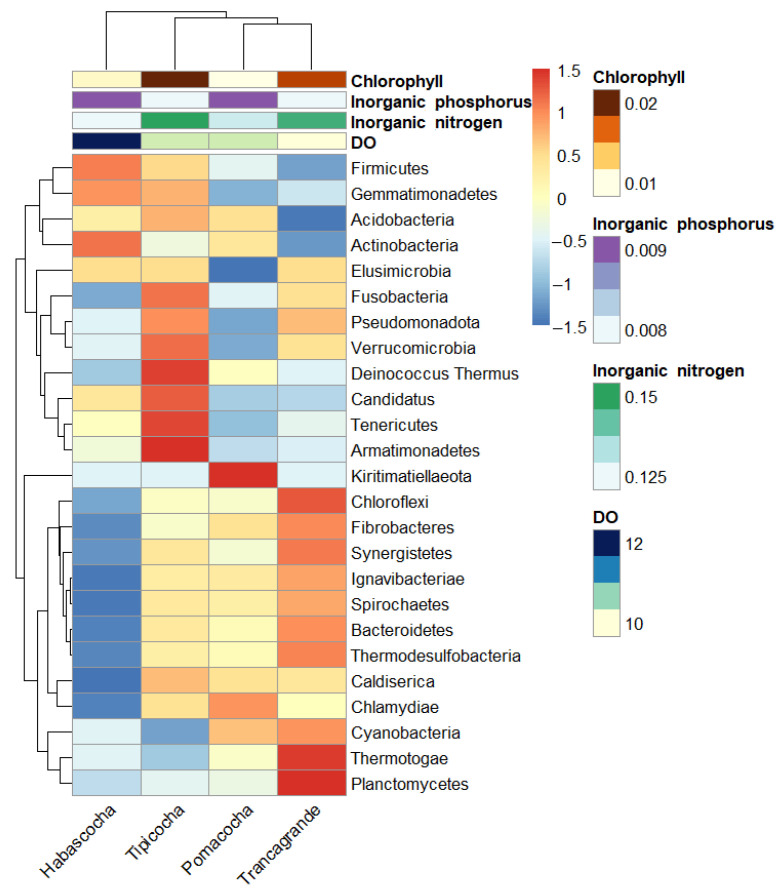
Heatmap of the relative abundance of dominant bacterial phylum annotated with environmental variables.

The heatmap (Figure 2) shows patterns in the relative abundance of dominant bacterial phyla across the four Andean lagoons. Pseudomonadota, Bacteroidetes, and Firmicutes exhibited higher relative abundance in Tipicocha and Trancagrande. Actinobacteria and Acidobacteria showed higher relative abundance in Habascocha.

Additional phyla such as Chloroflexi, Verrucomicrobia, Planctomycetes, and Gemmatimonadetes displayed variable enrichment across the sites. Hierarchical clustering indicated that Habascocha and Pomacocha shared similar phylum-level profiles, whereas Tipicocha differed more markedly from the other lagoons.

### 3.6. Correlation Networks Between Bacterial Functional Groups and Environmental Variables

**Figure 3:**
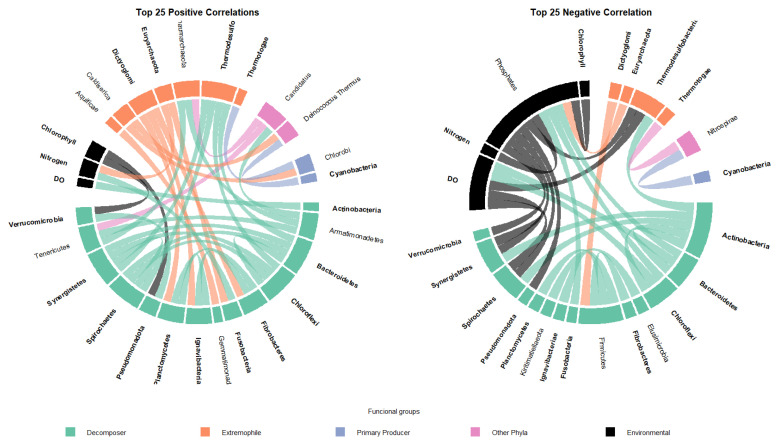
Spearman correlation network between bacterial functional groups and environmental variables.

A Spearman correlation analysis was performed to examine relationships between bacterial functional groups and environmental variables, retaining only statistically significant correlations (*p* < 0.05). From this subset, the 25 strongest positive and negative correlations were selected to visualize the dominant interaction patterns. The resulting network is shown in Figure 3.

The positive correlation network (left panel) displays a set of co-occurrences primarily involving the functional groups Decomposer and Extremophile, which showed multiple simultaneous positive associations with environmental variables. The negative correlation network (right panel) shows contrasting interactions, where the Environmental functional group appears as a central node with several negative correlations directed toward Decomposers. Environmental variables such as DO and inorganic nitrogen were among the most frequently connected parameters in both positive and negative networks (Figure 3).

Conversely, the negative correlation network (Figure 3, right panel) shows a contrasting interaction pattern, in which the Environmental functional group appears as a central node with multiple negative correlations directed toward groups classified as Decomposers. Additional negative associations were observed between functional groups related to primary production and decomposition processes. Environmental variables such as DO and inorganic nitrogen were among the most frequently connected factors in both the positive and negative networks, particularly in associations involving Pseudomonadota, Bacteroidetes, and Actinobacteria.

## 4. Discussion

The results reveal that the phylum Pseudomonadota dominated in the sediments of all the lagoons studied. The statistically significant gradient in TRIX (Table 2) and Chl-*a* across the lagoons acts as the primary ecological filter explaining the shift in microbial functional groups. This finding is consistent with reports from other high-altitude Andean water bodies under pressure from fish farming, where the high metabolic plasticity and tolerance to thermal variability of this phylum allow them to thrive in the central Andes [34]. Actinobacteria and Cyanobacteria also recorded notable abundances, a finding that aligns with studies in alpine lakes in Europe and Asia, where these groups thrive in conditions of high oxygen concentrations and UV radiation, respectively [35].

While phylum-level patterns outlined the dominant groups structuring the sediment microbiome, a finer ecological signal emerged at the class level. Therefore, the subsequent interpretation focuses on class-level responses, which revealed clearer associations with eutrophication, redox gradients and niche specialization.

A clear lagoon-specific pattern was observed when linking environmental conditions in the water column with sedimentary bacterial diversity. Tipicocha and Trancagrande, which showed the highest DIN (0.153 and 0.149 mg/L) and Chl-a (0.020 and 0.018 mg/L) values, also exhibited the greatest relative abundance of copiotrophic classes such as *Deltaproteobacteria* and *Gammaproteobacteria*. In contrast, Habascocha, the lagoon with the highest DO concentration (12.19 mg/L), presented the highest proportion of *Actinobacteria*, a group typically associated with oxygen-rich, oligotrophic sediments. Meanwhile, Pomacocha displayed intermediate conditions in both nutrients and diversity, matching the moderate distribution of dominant taxa. These results demonstrate a direct connection between eutrophication gradients in the water column and shifts in benthic microbial structure.

However, contrary to what is expected in eutrophic systems, the Tipicocha lagoon had the highest class-level Shannon and Simpson diversity indices. This suggests that its bacterial community is more even, which is likely due to the continuous input of organic matter from aquaculture, which fuels specialized degradation pathways and promotes microhabitat heterogeneity. This organic enrichment generates sharp redox gradients (hypoxia/anoxia) within the sediment, allowing the coexistence of both oligotrophic (aerobic) and copiotrophic/anaerobic (like Methanomicrobia) clades, thereby increasing overall microbial diversity and evenness [36]. This pattern contrasts with studies in Mediterranean marine farms, where diversity tends to decrease under cages due to strong organic enrichment.

To fully interpret this contrast, it is crucial to recognize the fundamental abiotic differences: the Mediterranean studies involve warm, high-salinity marine environments, whereas our Andean lagoons are characterized by low, cold temperatures, freshwater, and intense radiation. Despite these distinct physiochemical regimes, the convergence in microbial response, specifically the shift toward copiotrophic clades like Deltaproteobacteria and Gammaproteobacteria, underscores that organic enrichment acts as the universal, dominant selective pressure, overriding regional or thermal constraints [37]. The key difference lies in the outcome for diversity: in the oligotrophic Andean environment, the pulsed nutrient input initially creates new redox niches, leading to increased evenness; conversely, in already productive marine farms, organic enrichment simply intensifies existing anoxia, leading to competitive exclusion and decreased diversity. The partial similarity observed between the Habascocha and Pomacocha lagoons suggests that, despite their different feeding regimes, the high dissolved oxygen values in these environments mitigate the competitive exclusion that is common in highly eutrophic environments [38]. Compared to pristine high Andean lakes, where the Actinobacteria phylum dominates in cold, oligotrophic waters [39], aquaculture lagoons show a shift toward copiotrophic clades that could alter key biogeochemical cycles in the long term.

Although sediment geochemistry was not directly measured in this study, its mention aims to contextualize the observed microbial patterns within known redox and organic matter dynamics reported for similar Andean systems. Therefore, any inferences related to geochemical gradients are interpretative and should be verified in future work.

While the analysis successfully links the dominance of copiotrophic clades (Deltaproteobacteria, Gammaproteobacteria) to elevated inorganic nutrient and Chl-*a* levels, we acknowledge that this association is constrained by the limited number of environmental variables analyzed. Factors such as sediment organic matter content and temperature are known modifiers of the microbial structure, particularly in high-altitude environments. However, the consistent pattern across the lagoons strongly suggests that the anthropogenic nutrient load from fish farming acts as the primary, systemic causal factor. This load, although resulting in acceptable N and P concentrations in the water column, modulates secondary environmental variables shown in our analysis, specifically the DO gradient and the rate of primary production (Chl-*a*). The positive correlations between inorganic nitrogen, Chl-*a*, and the abundance of Deltaproteobacteria and Gammaproteobacteria (demonstrated in the Spearman analysis, Figure 3) support the hypothesis that the constant input of feed and excreta promotes bacterial communities specialized in degrading nitrogen compounds and particulate organic matter [40]. Specifically, Deltaproteobacteria includes numerous diazotrophs and sulfate reducers capable of thriving in ammonium-rich sediments with pronounced redox gradients [41]. Similar observations have been made in trout and sea bass cages, where increased fish biomass leads to a temporary increase in these clades [36]. For example, studies in Mediterranean aquaculture farms have shown that the enzymatic activity associated with Gammaproteobacteria increases significantly under the cages [11]. Furthermore, Fodelainakis et al. [42] demonstrated a clear spatial gradient of these bacterial groups, whose abundance decreases as the flow of particulate carbon from farming activity decreases. According to our SIMPER analysis, the aforementioned taxa explained more than 50% of the dissimilarity between the lagoons, reinforcing their role as sensitive indicators of the level of organic enrichment. Specifically, the elevated status of Tipicocha and Trancagrandewas directly linked to the enrichment of Flavobacteriia and Betaproteobacteria, bacterial groups associated with the hydrolysis of phytoplankton-derived polysaccharides [43]. This predictable enrichment suggest that bacterial composition responds quickly and predictably to organic load. This finding suggests that taxonomic monitoring can function as an early warning tool to optimize feed management and carrying capacity in aquaculture.

The positive relationship between dissolved oxygen and the abundance of Actinobacteria confirms observations in alpine lakes, where this phylum thrives in cold, well-oxygenated waters, serving as an indicator of oligotrophic conditions [38]. In the Habascocha lagoon, DO values (>12 mg/L) were associated with the highest proportion of Actinobacteria, while in Trancagrande, the reduction to less than 10 mg/L coincided with an increase in Deltaproteobacteria and the appearance of Methanomicrobia, indicating the formation of anoxic microenvironments [44]. This observed decline in Actinobacteria is thus strongly linked to the DO gradient, serving as the primary evidence for environmental filtering by the organic load, which increases oxygen consumption near the sediment. Recent river studies show that sediment oxygen demand can exceed 50% of total DO consumption in waters affected by organic matter [45]. This suggests that the accumulation of debris beneath the culture cages could create reducing niches even when the surface water column remains well oxygenated. The detection of Methanomicrobia and Bacilli is consistent with observations in sediments from intensive aquaculture ponds, where methanogenic and fermentative processes are intensified as a result of feces sedimentation [46]. These taxa, together with Euryarchaeota, could act as bioindicators of anoxia in early stages, before a critical drop in dissolved oxygen levels occurs. In a similar context, it has been proposed that the proliferation of sulfur-oxidizing bacteria, such as “cable bacteria,” in coastal systems could be a natural mechanism for mitigating sulfide accumulation [47]. Assessing the presence of these organisms in high Andean lakes could provide valuable information on the biogeochemical resilience of these ecosystems to ongoing organic enrichment.

The stabilization of communities dominated by copiotrophic clades suggests a potential increase in the rate of mineralization and the potential release of nutrients. If sustained, this cycle could feed back into the productivity of the lagoon and favor the appearance of toxic algal blooms, a phenomenon that has already been documented in Andean reservoirs related to climate change. However, establishing this direct causal link requires quantitative assessment of nitrogen and carbon transformation rates [2]. At the management level, the use of bacterial and archaeal consortia as bioindicators, a recommendation supported by recent reviews on benthic eDNA [48], would allow for the establishment of load thresholds based on microbial metrics that are more sensitive than classic physicochemical parameters. In addition, the implementation of fallowing or cage rotation programs could promote the recolonization of sediment by beneficial sulfur-oxidizing communities, as has been observed in marine ecosystems [47]. Our results, therefore, provide a crucial baseline for monitoring future changes in a context of glacial retreat and projected hydrological alterations in the central Andes [2]. However, the interaction between trace metals, temperature, and organic load still requires further experimental exploration, as high Andean sediments tend to accumulate arsenic and vanadium, elements known to modify microbial structure [44]. Future studies should incorporate direct measurements of sediment geochemical parameters (e.g., organic carbon, redox potential, and trace metal content) to quantify their influence on microbial community structure and validate the patterns inferred in this study. The consistent pattern across all lagoons clearly suggests that anthropogenic nutrient loading from fish farming acts as a primary and systemic causal factor. This load, although it results in acceptable concentrations of N and P in the water column, modulates the secondary environmental variables shown in the study data, specifically the DO gradient and the primary production rate (Chl-*a*).

## 5. Conclusions

This study demonstrated that fish farming activities in high-Andean lagoons are associated with changes in water trophic status and shifts in sedimentary bacterial communities. The integration of 16S rRNA sequencing with trophic indicators (DO, DIN, DIP, Chl-a and TRIX) confirmed that eutrophication gradients in the water column are directly reflected in the composition and structure of benthic microbiota. Copiotrophic and anaerobic groups dominated in lagoons with higher nutrient enrichment, whereas oxygen-rich systems retained taxa typical of oligotrophic conditions. These findings highlight the relevance of microbial communities as sensitive bioindicators of aquaculture-driven environmental change in Andean freshwater ecosystems. Future monitoring programs should incorporate microbial metrics to support sustainable management and prevent long-term ecological degradation.

## Figures and Tables

**Table 1:** Trophic Status Classification based on the TRIX Scale.

TRIX Range	Trophic Status	Water Quality	Description
<4	Oligotrophic	High	Low productivity and excellent water quality.
4 ≤ TRIX ≤ 5	Mesotrophic	Good	Moderate productivity, ecological balance.
5 < TRIX ≤ 6	Eutrophic	Moderate	High productivity, risk of imbalance.
>6	Hypertrophic	Poor	Excessive productivity and degradation of water quality.

**Table 2:** Physicochemical parameters and Trophic Index (TRIX) of water in the four lagoons.

Parameter	Habascocha	Pomacocha	Tipicocha	Trancagrande	EPA
	Mean ± SD	Mean ± SD	Mean ± SD	Mean ± SD	
DO (mg/L)	12.193 ± 0.686 ^a^	10.375 ± 1.109 ^b^	10.350 ± 1.066 ^b^	9.850 ± 0.597 ^c^	≥5.0 mg/L
Inorganic nitrogen (mg/L)	0.122 ± 0.010 ^a^	0.128 ± 0.009 ^a^	0.153 ± 0.017 ^b^	0.149 ± 0.003 ^b^	≤0.3 mg/L
Inorganic phosphorus (mg/L)	0.009 ± 0.002 ^a^	0.009 ± 0.001 ^a^	0.008 ± 0.001 ^a^	0.008 ± 0.001 ^a^	≤0.05 mg/L
Chl-*a* (mg/L)	0.011 ± 0.002 ^a^	0.010 ± 0.003 ^a^	0.020 ± 0.001 ^b^	0.018 ± 0.001 ^b^	
**Trophic Index**					
TRIX	5.463 ± 0.099 ^a^	5.541 ± 0.074 ^a^	5.739 ± 0.081 ^b^	5.678 ± 0.071 ^b^	

Note: EPA: Environmental protection agency. Values are presented as Mean ± SD, based on n = 9 measurements per lagoon. Different superscript letters (^a^, ^b^, ^c^) indicate statistically significant differences between lagoons (one-way ANOVA followed by Tukey’s HSD post hoc test, *p* < 0.05).

**Table 3:** Normalized read counts of bacterial phyla identified in each lagoon.

Phylum	Habascocha	Pomacocha	Tipicocha	Trancagrande
Acidobacteria	2668	2901	3284	1023
Actinobacteria	20,234	13,410	8668	3407
Aquificae	83	1	19	7
Armatimonadetes	23	15	66	18
Bacteroidetes	7001	10,302	10,980	12,591
Caldiserica	20	76	87	74
Candidatus	507	328	652	337
Chlamydiae	15	110	81	61
Chlorobi	14	69	142	51
Chloroflexi	1740	2234	2260	2964
Chrysiogenetes	1	0	0	0
Crenarchaeota	0	0	0	1
Cyanobacteria	13,986	20,855	10,245	22,762
Deferribacteres	0	4	4	0
Deinococcus Thermus	190	335	653	250
Dictyoglomi	112	758	733	1097
Elusimicrobia	2	1	2	2
Euryarchaeota	319	2534	2837	2668
Fibrobacteres	12	32	25	41
Firmicutes	8616	5975	7613	4841
Fusobacteria	35	43	65	55
Gemmatimonadetes	2008	407	1818	654
Ignavibacteriae	442	1440	1418	1876
Kiritimatiellaeota	2	3	2	2
Nitrospirae	653	116	1761	82
Planctomycetes	45	59	55	148
Pseudomonadota	58,539	55,169	66,426	64,971
Spirochaetes	108	447	469	612
Synergistetes	16	38	52	74
Tenericutes	147	116	198	134
Thaumarchaeota	7	2	66	3
Thermodesulfobacteria	173	329	353	459
Thermotogae	14	15	13	19
Verrucomicrobia	636	516	969	814
Total Normalized Reads	91,704	86,079	98,700	85,394

Note: Numerical values represent the total number of quality-filtered 16S rRNA gene sequence reads assigned to each phylum, normalized across all samples. The value of ‘0’ indicates that the taxon was not detected in the sequencing run for that specific lagoon, rather than missing data.

**Table 4:** Diversity index of bacterial class in each lagoon.

Index	Habascocha	Pomacocha	Tipicocha	Trancagrande
Richness (S)	65	62	63	62
Dominance (D)	0.1135	0.1101	0.1031	0.1124
Shannon diversity (H′)	2.627	2.664	2.769	2.658
Simpson diversity (1-D)	0.8865	0.8899	0.8969	0.8876
Estimated richness (Chao-1)	66	67	64.5	65

Note: The indices shown quantify different aspects of alpha diversity at the class level. Total abundance refers to the sum of normalized sequence reads for all classes in each lagoon. Richness (S) represents the number of distinct bacterial classes. Dominance (D) measures the extent to which one or a few classes overwhelmingly contribute to abundance. Shannon diversity (H′) incorporates both richness and evenness, whereas Simpson diversity (1-D) gives more weight to dominant classes. Estimated richness (Chao-1) predicts the total number of classes, including those potentially underrepresented in the sequencing dataset.

**Table 5:** Contribution (%) of bacterial classes to differences in the microbial composition of lagoons (SIMPER).

Comparison	Bacterial Class	Contribution (%)	Mean Abundance	Cumulative Contribution
Habascocha vs. Pomacocha	Deltaproteobacteria	18.2	16.8	18.2
	Alphaproteobacteria	18.1	13.2	36.3
	Actinobacteria	9.2	11.9	45.5
	Gammaproteobacteria	8.5	9.2	54
	Flavobacteriia	7.4	4.7	61.4
	Betaproteobacteria	6.6	14.5	68
Habascocha vs. Tipicocha	Actinobacteria	23.4	10.2	23.4
	Betaproteobacteria	13.3	14.1	36.7
	Deltaproteobacteria	13	12.7	49.7
	Gammaproteobacteria	9.5	8.1	59.3
	Flavobacteriia	5.3	3.1	64.6
	Methanomicrobia	5	1.3	69.6
Habascocha vs. Trancagrande	Actinobacteria	20.3	8.8	20.3
	Deltaproteobacteria	12.6	13.9	33
	Gammaproteobacteria	9.3	9	42.3
	Alphaproteobacteria	9.2	17.1	51.5
	Flavobacteriia	8.3	4.6	59.8
	Methanomicrobia	3.6	1.4	63.5
	Bacilli	2.9	2.3	66.4
	Betaproteobacteria	2.7	17.9	69.1

## Data Availability

The data that support the findings of this study are available from the corresponding author upon reasonable request.

## Abbreviations

The following abbreviations are used in this manuscript:

DO	Dissolved Oxygen
Chl-*a*	Chlorophyll-*a*
DIN	Dissolved Inorganic Nitrogen
DIP	Dissolved Inorganic Phosphorus
TRIX	Trophic Index
DNA	Deoxyribonucleic Acid
eDNA	Environmental DNA
PCR	Polymerase Chain Reaction
NGS	Next-Generation Sequencing
SIMPER	Similarity Percentage Analysis
SIMPROF	Similarity Profile Analysis
